# Multispectral co-occurrence of wavelet coefficients for malignancy assessment of brain tumors

**DOI:** 10.1371/journal.pone.0250964

**Published:** 2021-06-17

**Authors:** Shaswati Roy, Pradipta Maji

**Affiliations:** 1 Department of Information Technology, RCC Institute of Information Technology, Kolkata, West Bengal, India; 2 Biomedical Imaging and Bioinformatics Lab, Machine Intelligence Unit, Indian Statistical Institute, Kolkata, West Bengal, India; University of Oklahoma, UNITED STATES

## Abstract

Brain tumor is not most common, but truculent type of cancer. Therefore, correct prediction of its aggressiveness nature at an early stage would influence the treatment strategy. Although several diagnostic methods based on different modalities exist, a pre-operative method for determining tumor malignancy state still remains as an active research area. In this regard, the paper presents a new method for the assessment of tumor grades using conventional MR sequences namely, T1, T1 with contrast enhancement, T2 and FLAIR. The proposed method for tumor gradation is mainly based on feature extraction using multiresolution image analysis and classification using support vector machine. Since the wavelet features of different tumor subregions, obtained from single MR sequence, do not carry equally important information, a wavelet fusion technique is proposed based on the texture information content of each voxel. The concept of texture gradient, used in the proposed algorithm, fuses the wavelet coefficients of the given MR sequences. The feature vector is then derived from the co-occurrence of fused wavelet coefficients. As each wavelet subband contains distinct detail information, a novel concept of multispectral co-occurrence of wavelet coefficients is introduced to capture the spatial correlation among different subbands. It enables to convey more informative features to characterize the tumor type. The effectiveness of the proposed method is analyzed, with respect to six classification performance indices, on BRATS 2012 and BRATS 2014 data sets. The classification accuracy, sensitivity, specificity, positive predictive value, negative predictive value, and area under curve assessed by the ten-fold cross-validation are 91.3%, 96.8%, 66.7%, 92.4%, 88.4%, and 92.0%, respectively, on real brain MR data.

## 1 Introduction

Brain tumor is not very common, but it is among the most fatal cancers [1]. It can be defined as an abnormal lump of tissues, which infiltrates surrounding brain tissues and interferes the normal brain activities. The treatment strategy for the patients with brain tumor depends on the assessment of its malignancy state. According to its aggressiveness, the World Health Organization (WHO) classifies brain tumor into grades I to IV. The patients with high grade tumor, classified as either WHO grade III or grade IV, have a worse prognosis, with median survival rate of about two years or less. Glioblastoma multiforme (WHO grade IV) is the most common malignant brain tumor. It develops very rapidly, yielding poor median survival rate of about 14 months. Therefore, the patients with high grade brain tumor require aggressive treatment, such as chemotherapy and radiation, as soon as possible. In contrast, the patients with low grade brain tumor, classified as either WHO grade I or grade II, have a better life expectancy of several years. In effect, aggressive treatment can be delayed in this case. Thus, the accurate classification of brain tumor into high and low grades is important to determine the treatment strategies.

Many techniques have been proposed so far to quantify the tumor heterogeneity as an imaging biomarker for the prediction of tumor grades [2]. Magnetic resonance imaging (MRI) is an important diagnostic technique for non-invasively providing accurate information about the existence, extent, and aggressiveness of brain tumor. The MR images give the anatomical and structural information, not only about the active brain tumor area, but also about the surrounding tissues, which is helpful in characterizing the tumor grade. Dean et al. [3] have showed the importance of MR images in a group of patients with gliomas, and verified the analysis with the corresponding results of biopsy diagnosis. In fact, MRI provides different sequences such as T1-weighted (T1), T1-weighted with contrast enhancement (T1C), T2-weighted (T2), and fluid-attenuated inversion recovery (FLAIR), to capture different types of tissue contrasts. The T1 sequence allows structural analysis of healthy tissues, while tumor borders appear brighter in T1C. Moreover, the necrotic and active tumor areas can be easily discriminated in the T1C MR sequence. In T2 sequence, the edema region appears brighter, whereas it can be separated properly from cerebrospinal fluid in FLAIR. Therefore, one MRI sequence is not sufficient to characterize all the subregions of brain tumors. In effect, a combination of these sequences provides a considerable amount of information for revealing the underlying tumor grade. In [4], a fusion strategy is proposed to combine the structural and textural information of these four MRI sequences for the detection of brain tumor. A fused single image has been reconstructed using the inverse discrete wavelet transform from the coefficient matrices of the MRI sequences. Cheng et al. [5] proposed a fusion technique to automatically learn a mapping function for integrating multimodal features. A weight map is learnt to gate the scale of information. The gated content from individual MRI modalities is then fused to form the integrated representation.

In a brain MR image, texture analysis provides information about the uniformity of image intensities. The textural properties of brain tumor region give valuable information for the prediction of tumor type. The wavelet based multiresolution analysis [6] is the most effective technique for extracting the textural features from brain MR images [7–9]. In [7], the wavelet analysis is performed on apparent diffusion coefficient images to predict the degree of malignancy of brain tumor. In [10], brain tumor texture is formulated using a multiresolution-fractal model for characterizing patient-independent brain tumor texture. Bauer et al. [11] have presented an approach to establish the correspondence between a healthy atlas and MR images of tumor patients. A tumor growth model, based on multiscale-multiphysics model, in combination with registration algorithms, has been employed. Another approach for extracting textural information is the computation of gray level co-occurrence matrix (GLCM). The GLCM considers the gray level transition between two voxels, and several features can be extracted from the GLCM. In order to increase the discriminability of the descriptor, several extensions of GLCM are proposed in the literature [12–16]. Siqueira et al. [12] extended the GLCM to multiple scales through two different approaches namely, a Gaussian scale-space representation and an image pyramid. Gelzinis et al. [13] computed the co-occurrence matrices simultaneously for different distance parameter values. In [14], multi-scale texture analysis using GLCM is introduced by changing the windows size. Pacifici et al. [15] performed GLCM based multi-scale texture analysis using multiple windows with different directions and different shift values of displacement vector. Walker et al. [16] extracted features from the GLCM by weighted sum of the elements of these matrices in localized neighborhoods. In [17], the distance parameter is varied from one to six to generate the multi-scale GLCM.

Law et al. [18] showed that relative cerebral blood volume measurements, derived from perfusion MR imaging and metabolite ratios from proton MR spectroscopy, are useful in predicting glioma grade. In this case, the histopathologic grading has been used to verify the tumor grade determined from the method. Li et al. [19] used patient specific fifteen features and support vector machine (SVM) with floating search method to predict the degree of malignancy of brain tumor. However, the selection of these patient specific features requires the intervention of domain experts. In [7], a mixture of unsupervised artificial neural networks and hierarchical multiresolution wavelet has been used to evaluate the degree of aggressiveness of brain tumor. In this work, the wavelet filtered apparent diffusion coefficient images, along with T2 and FLAIR images, are used to generate the features. Zacharaki et al. [20] used a combination of conventional MRI and perfusion MRI to extract the features, followed by feature selection and classification. The features include tumor shape and intensity characteristics as well as rotation invariant texture features. The SVM, with recursive feature elimination, is used to obtain the feature subset. In [21], an unsupervised method is proposed to obtain clustered images from diffusion tensor images using multiple parameters. A two-level clustering approach namely, self-organizing map followed by *k*-means algorithm, has been developed to enable unsupervised clustering of images. These clustered images then allow visual grading of gliomas by applying the SVM. To determine the glioma grade, the 16-class diffusion tensor-based clustered images are used. In [22], tumor heterogeneity is evaluated by using texture analysis performed on apparent diffusion coefficient maps. Three features are extracted within the region of interest. These include entropy, obtained from gray level co-occurrence matrices, and the skewness and kurtosis of the image histogram. These texture and histogram features act as the parameters to discriminate between low and high grade gliomas using an unpaired student’s *t*-test.

Therefore, the pattern recognition methods, in a supervised manner, are useful for the prediction of tumor grades. However, the performance of the existing methods is mainly affected by two factors: 1) variability in information of different MR sequences; and 2) identification of proper descriptors that can capture the intrinsic textural properties for proper tumor grading. Moreover, the brain tumor gradation methods, reported recently in [7, 20–22], have used several high-cost advanced modalities, such as diffusion-weighted images and perfusion MRI. A brain tumor gradation method that relies on easily accessible low-cost conventional MR images is desirable.

In this regard, a new method is proposed for the assessment of tumor grades. The proposed algorithm projects the tumor region onto an appropriate feature space, which is able to capture the essential attributes for differentiating different tumor types. Given several low-cost MR sequences namely, T1, T1C, T2 and FLAIR, the proposed algorithm combines the textural features from these images. The multiresolution wavelet analysis is performed to extract the image features within the region of interest. The proposed method introduces a fusion algorithm that combines the wavelet coefficients of the MR sequences, depending on the texture information content of different tumor subregions. After generating the fused wavelet subbands, feature vector is obtained from the co-occurrence of wavelet coefficients. In order to capture the spatial correlation among different wavelet subbands, a novel concept called multispectral co-occurrence of wavelet coefficients is introduced in this paper. The proposed method, for computing the co-occurrence of a pair of wavelet coefficients, can capture the edge continuity present along different subbands. Subsequently, seven Haralick textural features are computed from the proposed multispectral co-occurrence matrix of wavelet coefficients, which are then fed into the SVM to classify the tumor types. Finally, the effectiveness of the proposed method is analyzed using leave-one-out cross-validation and ten-fold cross-validation strategies, along with a comparison with other methods.

## 2 Proposed methodology

**Fig 1:**
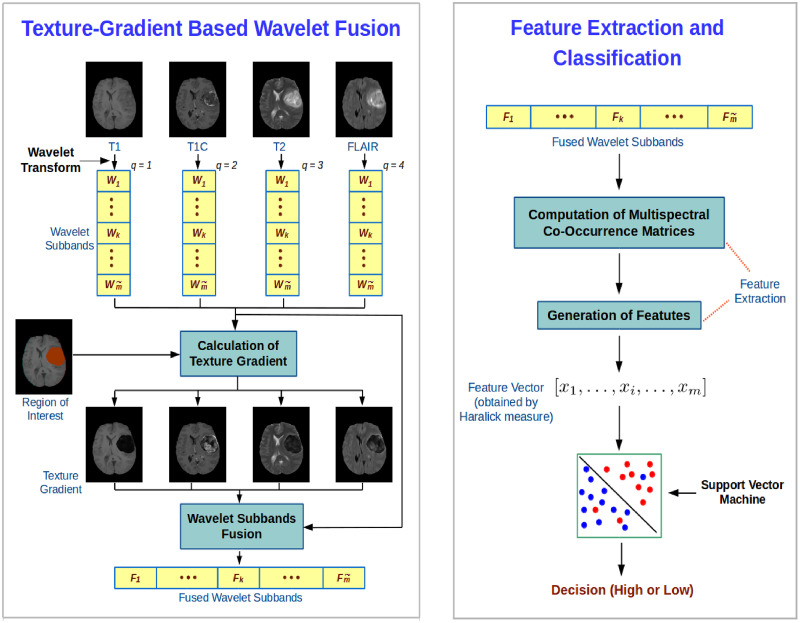
Block diagram of the proposed brain tumor gradation method.

This section presents a novel method for the evaluation of malignancy stage of brain tumor using MR images. The overview of the processing pipeline used in this study is depicted in Fig 1. It consists of mainly three steps as described below:

1. Wavelet fusion of different MR sequences based on texture gradient;
2. Generation of feature vector obtained from multispectral co-occurrence matrix of fused wavelet coefficients; and
3. Gradation of tumor subtypes using the SVM.

The first step is mentioned in left block of Fig 1, while the remaining two steps are presented in right block of this figure. Each step of the proposed tumor gradation algorithm is elaborated next one by one.

### 2.1 Texture-gradient based wavelet fusion

Image fusion is often desirable to merge images having complementary characteristics, so that essential aspects of each image can be retained. The variability in information of different MR sequences makes fusion as an important step for determining the tumor type. In [7], it has been shown that the texture analysis can appropriately capture the characteristics of individual regions of an entire tumor. The multiresolution approach of wavelet is suitable for extracting local textural features from the images. Since every wavelet coefficient carries unique information, different textural information can be used to identify different homogeneous texture regions within a tumor region, such as necrosis, enhancing, non-enhancing, and edema. The association among these regions can reveal the tumor characteristics. Therefore, the texture gradient [23], which can capture the differences in textures within a tumor region, can be used suitably in characterizing brain tumor.

In this background, a new wavelet based fusion method (left block of Fig 1) is proposed. It employs texture content of each spatial position of each resolution level to fuse the images. The wavelet decomposition generates detail subbands, containing important high frequency components at different directions. In the proposed method, the detail information is extracted from each of the four MR sequences namely, T1, T1C, T2 and FLAIR, using 3-D dyadic wavelet decomposition. However, the features of different tumor subregions, obtained from one MR sequence, do not carry equally important information. In other words, one tumor subregion may contain more detail information in one MR sequence, whereas the same tumor subregion may have less textural information in another sequence. Therefore, it is reasonable to characterize the texture content of each spatial position at each spectrum to obtain most desirable information. In this regard, the proposed wavelet based fusion algorithm considers texture gradient within tumor region to fuse the given MR images.

Let, the input brain MR volume be decomposed by dyadic wavelet upto *L*th level. So, the number of generated detail subbands is $\tilde{m}=d\times L$. Here, *d* is the number of detail subbands generated at each decomposition level. In case of 3-D dyadic wavelet decomposition, used in the current study, the value of *d* is 7. Let, *P* be the number of MR sequences analyzed and $W_{q}^{p}$ denotes each of the wavelet detail subbands, where $q=1,\ldots ,\tilde{m}$ and *p* = 1, …, *P*. Let, **r** = (*x*, *y*, *z*) be a coordinate vector in $W_{q}^{p}$ and *G*^*p*^(**r**) be the texture gradient at position **r** for the *p*th sequence. The proposed fusion method integrates the detail images of each resolution level *q* and at each position **r** of *q* as follows:

$$\begin{array}{c} F_{q}\left(r\right)=\sum_{p=1}^{P}G^{p}\left(r\right)⋆W_{q}^{p}\left(r\right); \end{array}$$ (1)

where ⋆ represents point-wise multiplication and *F*_*q*_(**r**) denotes the *q*th detail subband with fused information at spatial position **r**. In the proposed fusion method, the magnitude of texture gradient gives the weight value for corresponding MR sequence in fusion. In this way, the regions with more texture content contribute more in fusion, thereby, maximizing the information flow from subbands of individual sequence to fused wavelet subbands. In order to compute the texture gradient for each MR sequence, median filtering, with a kernel of size 3 × 3 × 3, is performed on each $W_{q}^{p}$, followed by gradient extraction. The texture gradient, termed as *G*^*p*^(**r**), is then derived by adding the gradient magnitudes of each subband as follows:

$$\begin{array}{c} G^{p}\left(r\right)=\overset{\tilde{m}}{\underset{q=1}{\sum }}\frac{\left|\nabla M\left(W_{q}^{p}\left(r\right)\right)\right|}{l_{2}\left(M\left(W_{q}^{p}\right)\right)},p=1,\ldots ,P; \end{array}$$ (2)

where $M(W_{q}^{p})$ represents the median filtered subband of $W_{q}^{p}$ and ∇ is approximated using gradient extraction technique. The normalizing term $l_{2}\left(M\left(W_{q}^{p}\right)\right)$ denotes *L*_2_-norm energy of median filtered subband of $W_{q}^{p}$.

### 2.2 Multispectral co-occurrence of wavelet coefficients

**Fig 2:**
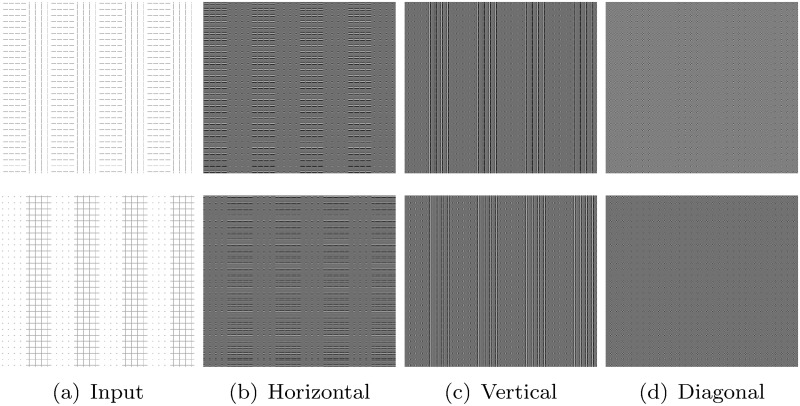
Detail wavelet subbands of two simulated images having different textures.

**Fig 3:**
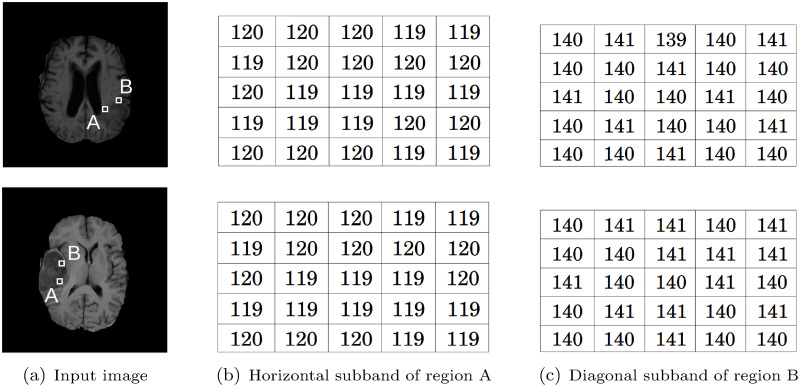
Detail wavelet subbands of two T1-weighted brain MR images with high grade (top) and low grade (bottom) tumors.

Texture analysis plays an important role in measuring the aggressiveness of brain tumor. The GLCM is one of the dominant texture descriptors used in image analysis. It has been extended in [12–16] to capture texture information of the given image at multiple scales. In these approaches, features are extracted from each co-occurrence matrix of multiple scales and then concatenated to obtain the final feature vector. Although the concatenation of features obtained from co-occurrence matrices of different wavelet subbands is able to capture texture information at multiple scales [17], it has the drawback that each co-occurrence matrix, representing the relationships of wavelet coefficients, is confined into a single subband. Fig 2(a) presents two simulated images having different textures, while Fig 3(a) presents T1-weighted brain MR images with high grade and low grade tumors. The image at the top row of Fig 2(a) contains vertical and horizontal lines, while the image of bottom row of Fig 2(a) contains criss-cross lines and dotted region. Fig 2(b)–2(d) present the detail subbands resulting from the wavelet decomposition of two simulated images of Fig 2(a). For brain tumor images, the horizontal and diagonal subbands are presented in Fig 3(b) and 3(c) for two different tumor regions namely, A and B, respectively. Analyzing the detail subbands in Fig 2(b)–2(d), it is observed that the vertical and diagonal subbands of the two simulated images of Fig 2(a) are similar. In case of brain tumor images presented in Fig 3, the wavelet coefficients of horizontal subbands for region A, computed from high grade and low grade brain tumor images, are almost same. Similarly, the wavelet coefficients of diagonal subbands for region B of these two brain tumor images are nearly equal to each other. Therefore, it is seen that the co-occurrence of wavelet coefficients computed within a single subband, such as vertical and diagonal in Fig 2 and horizontal and diagonal in Fig 3, cannot reflect well the distinction present in the patterns of these images.

In this regard, the proposed algorithm introduces a novel concept, called multispectral co-occurrence matrix of wavelet coefficients. It considers the joint distribution of wavelet coefficient pairs of neighboring points that are taken, not only from single spectrum, but also from multiple spectra of different resolution levels. In effect, the features, derived from the proposed multispectral co-occurrence matrix of wavelet coefficients, have a high descriptive ability as well as retain the advantage of co-occurrence of wavelet coefficients. Each element of the proposed multispectral co-occurrence matrix represents the number of transitions between each pair of intra- and inter-subband wavelet coefficients involved in the spatial relationship denoted by $\Delta \tilde{r}$. So, each (*i*, *j*)-th element of multispectral co-occurrence matrix for the *q*th subband is defined as follows:

$$\begin{array}{c} P_{q}\left(i,j,\Delta \tilde{r}\right)=\#\{(r,q),(r+\Delta \tilde{r},\tilde{q})\;|\;F_{q}\left(r\right)=i,\;F_{\tilde{q}}\left(r+\Delta \tilde{r}\right)=j\}; \end{array}$$ (3)

where $q=1,\ldots ,\tilde{m}$, and # denotes the number of elements in the set.

The parameter $\Delta \tilde{r}=\left(dx,dy,dz\right)$ is a displacement vector between the pair of wavelet coefficients and $\tilde{q}$ ($=1,\ldots ,\tilde{m}$) denotes any spectrum. Here, the term (**r**, *q*) represents the location of a wavelet coefficient which resides at spatial position **r** of *q*th detail wavelet subband. The computation of multispectral co-occurrence matrix for an example 4 × 4 matrix is explained in Appendix 5.1.

**Fig 4:**
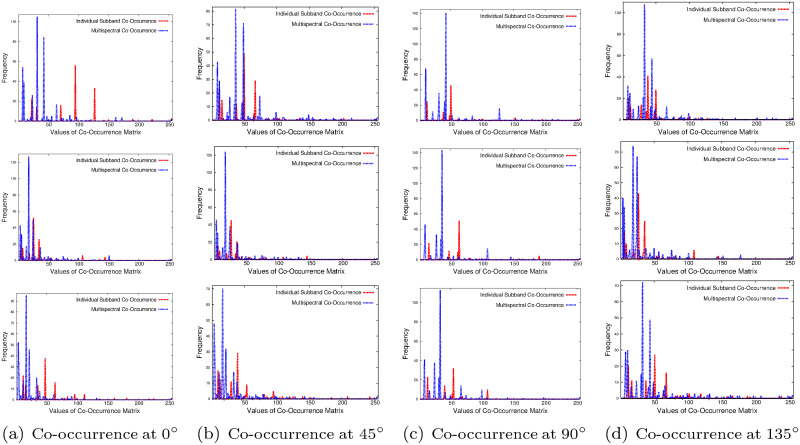
Histograms of co-occurrence of wavelet coefficients for different subbands (top row: Horizontal, middle row: Vertical, and bottom row: Diagonal) of top image of Fig 2.

**Fig 5:**
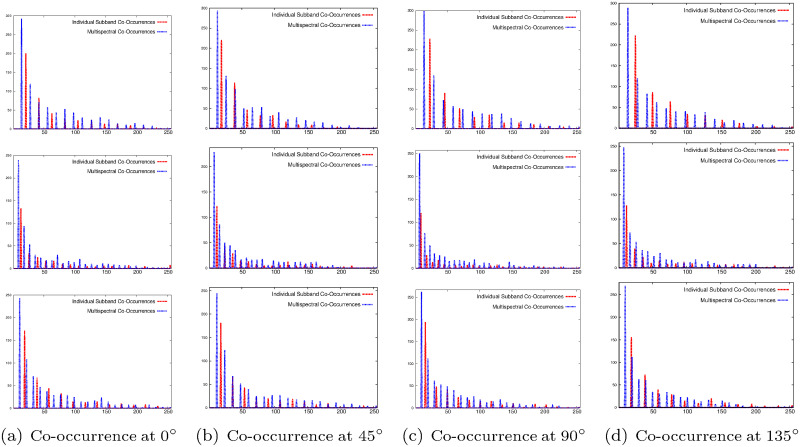
Histograms of co-occurrence of wavelet coefficients for different subbands (top row: Horizontal, middle row: Vertical, and bottom row: Diagonal) of top image of Fig 3.

**Fig 6:**
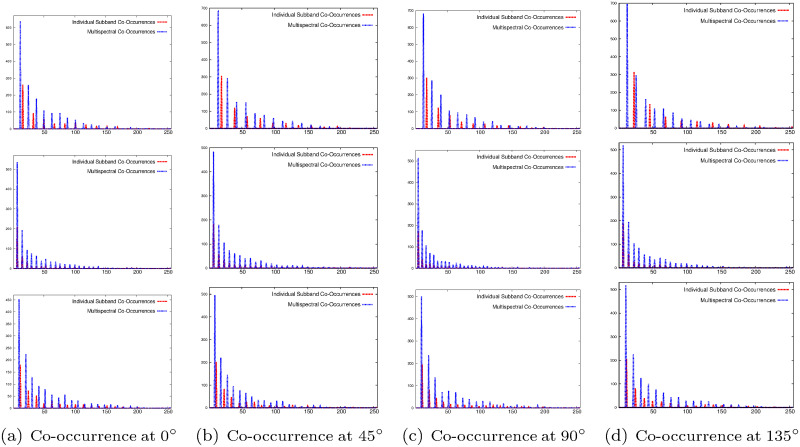
Histograms of co-occurrence of wavelet coefficients for different subbands (top row: Horizontal, middle row: Vertical, and bottom row: Diagonal) of bottom image of Fig 3.

The importance of the proposed multispectral co-occurrence matrices over the co-occurrence matrix computed from individual wavelet subbands is illustrated in Figs 4–6 and Tables 1 and 2. Figs 4–6 present the histograms of co-occurrence matrices obtained from different wavelet subbands of top image of Fig 2(a) and two brain tumor images of Fig 3(a), considering single subband, termed as “Individual Subband Co-Occurrence” and multiple subbands, termed as “Multispectral Co-Occurrence”. The co-occurrence of wavelet coefficients of the example images is measured at 0°, 45°, 90°, and 135° directions for the computation of both individual subband and multispectral co-occurrence matrices, as shown in Figs 4–6. Analyzing the histograms presented in Figs 4–6, it is seen that the number of distinct pairs of wavelet coefficients obtained from multispectral co-occurrence matrix is significantly higher than that obtained from single subband co-occurrence matrix. The wavelet transform analyzes the image at a nested set of scales at three different directions namely, horizontal, vertical and diagonal. Each wavelet coefficient represents image contents localized in spatial location and frequency, which enables wavelet transform to efficiently represent the local edge contents of the image for different orientations. The proposed multispectral co-occurrence matrix considers the wavelet coefficients of different wavelet spectra. Therefore, it is able to capture the correlation of important image content located at all higher frequency components of the image, which results in generating significantly more number of distinct pairs of wavelet coefficients than that obtained from the co-occurrence matrix computed within single subband, as shown in Figs 4–6. Hence, the features derived from the multispectral co-occurrence matrix can adequately capture the edge continuity among different subbands, while the co-occurrence matrix of a single subband losses important spatial information.

**Table 1:** Textural features obtained from individual subband co-occurrence and multispectral co-occurrence for two example images.

Different Features	Different Subbands	Individual Subband Co-Occurrence			Multispectral Co-Occurrence		
		Image 1	Image 2	Difference	Image 1	Image 2	Difference
Inverse Difference Moment	Horizontal	0.504808	0.486144	0.018664	0.179289	0.212561	0.033272
	Vertical	0.475911	0.472579	0.003332	0.293663	0.326703	0.033040
	Diagonal	0.572053	0.562862	0.009191	0.206421	0.268635	0.062214
Sum Average	Horizontal	184.157043	184.09903	0.058013	189.389069	184.712952	4.676117
	Vertical	184.074371	184.128784	0.054413	189.369431	184.772278	4.597153
	Diagonal	205.200027	187.953094	17.246933	215.733109	186.681305	29.051804
Correlation	Horizontal	-0.18593	-0.186809	0.000879	0.061935	0.070602	0.008667
	Vertical	-0.17954	-0.179366	0.000174	-0.067012	-0.064276	0.002736
	Diagonal	-0.127586	-0.127515	0.000071	-0.018895	-0.020897	0.002002

**Table 2:** Textural features obtained from individual subband co-occurrence and multispectral co-occurrence for brain images with high and low grade tumors.

Different Features	Different Subbands	Individual Subband Co-Occurrence			Multispectral Co-Occurrence		
		High Grade	Low Grade	Difference	High Grade	Low Grade	Difference
Inverse Difference Moment	Horizontal	0.156010	0.133686	0.022324	0.095357	0.051982	0.043375
	Vertical	0.252172	0.245452	0.006720	0.124291	0.082882	0.041409
	Diagonal	0.163154	0.172801	0.009647	0.118987	0.064271	0.054716
Entropy	Horizontal	5.876775	6.164102	0.287327	6.391913	7.070074	0.678161
	Vertical	5.114573	5.346992	0.232419	5.981598	6.625370	0.643772
	Diagonal	5.618186	5.722505	0.104319	6.216316	6.797121	0.580805
Sum Entropy	Horizontal	3.452767	3.586325	0.133558	3.664164	4.332994	0.668830
	Vertical	3.086843	3.247257	0.160414	3.571429	4.229835	0.658406
	Diagonal	3.236954	3.277212	0.040258	3.606415	4.250120	0.643705

The advantage of the proposed multispectral co-occurrence matrix over the co-occurrence matrix computed from individual wavelet subband is also illustrated quantitatively using Haralick features in Tables 1 and 2. Three Haralick features namely, inverse difference moment, sum average and correlation, are examined for two simulated images of Fig 2(a), while a different set of three Haralick features namely, inverse difference moment, entropy and sum entropy is considered for two brain tumor images of Fig 3(a). These features are computed for each wavelet subband, obtained from different co-occurrence matrices, for two simulated images of Fig 2(a) and for two brain tumor images of Fig 3(a), and the corresponding results are presented in Tables 1 and 2, respectively. Analyzing the feature values reported in these tables, it is seen that the values of Haralick features are almost similar for both simulated images as well as for high grade and low grade brain tumor images at each wavelet subband when the co-occurrence matrix is computed within single subband. On the other hand, the difference between feature values for the two simulated images and two brain tumor images is significantly higher when considering the multispectral co-occurrence matrices, as shown in Tables 1 and 2, respectively. Therefore, the features computed from the co-occurrence matrices of individual subbands cannot adequately discriminate between two types of patterns present in these simulated and brain tumor images and thus become insignificant for further analysis.

The proposed multispectral co-occurrence matrices at different orientations can be formed by using different displacement vectors as mentioned in (3). In the volumetric analysis, 26 different combinations of the displacement vectors $\Delta \tilde{r}$ or the spatial relationship can be obtained; and among them 13 are distinct (mentioned in Appendix 5.2). For the simplification of computation, it is usually set as one voxel in distance and it, thus, yields only 13 different types of configurations of wavelet coefficient pairs in 3-D space. In the current study, the coefficients of each wavelet subband are normalized in the range of 0 to 255 before computing the multispectral co-occurrence matrices.

In order to extract useful information present in co-occurrence matrices, the proposed algorithm forms a feature vector for each subband *q*. The feature set includes seven Haralick measures namely, contrast, correlation, inverse difference moment, sum average, sum variance, sum entropy, and difference variance. The resultant feature vector for each subband is then obtained by averaging the feature vectors obtained from co-occurrence matrices for 13 different orientations. Next, the final feature vector is derived by concatenating the averaged feature vectors, obtained from the proposed multispectral co-occurrence matrices of each subband. So, the dimension of the feature vector becomes $m=7\times \tilde{m}$, as seven Haralick features are used in the current study.

### 2.3 Gradation of brain tumor

In the present work, support vector machine (SVM) [24] is used to classify low grade and high grade brain tumors. The SVM is a margin classifier, defined by an optimal hyperplane in the feature vector space. For a given labeled training data, the SVM outputs a decision hyperplane which categorizes new query instances. An important property of the SVM is that it is robust to outliers. It finds a decision boundary that maximizes the margin between two classes and tolerates the individual outliers at the same time. Moreover, the SVM is able to construct nonlinear decision boundary using kernel tricks. In the current study, linear kernels are used.

## 3 Experimental results and discussions

The performance of the proposed algorithm for evaluating the brain tumor malignancy is extensively studied and compared with that of some existing algorithms. The classification performance is assessed using two strategies namely, ten-fold cross-validation and leave-one-out cross-validation. The performance of the proposed method is analyzed with respect to six quantitative indices namely, accuracy, sensitivity, specificity, positive predictive value (PPV), negative predictive value (NPV) and area under the receiver operating characteristic (ROC) curve (AUC). The definitions of these indices are presented in Appendix 5.3. For ten-fold cross-validation, the mean value of each index, computed over ten-folds, is reported. On the other and, for leave-one-out cross-validation, the decision value is evaluated for each and every test subject, and after all repeats, these decision values are used to compute the classification performance indices.

**Fig 7:**
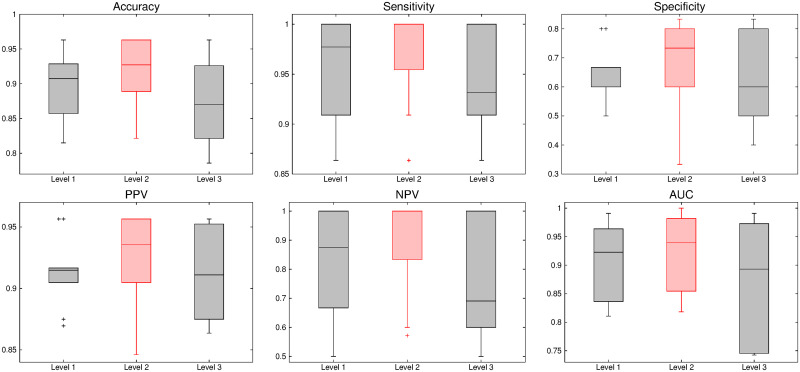
Comparative performance analysis of different decomposition levels of wavelet analysis using ten-fold cross-validation.

**Fig 8:**
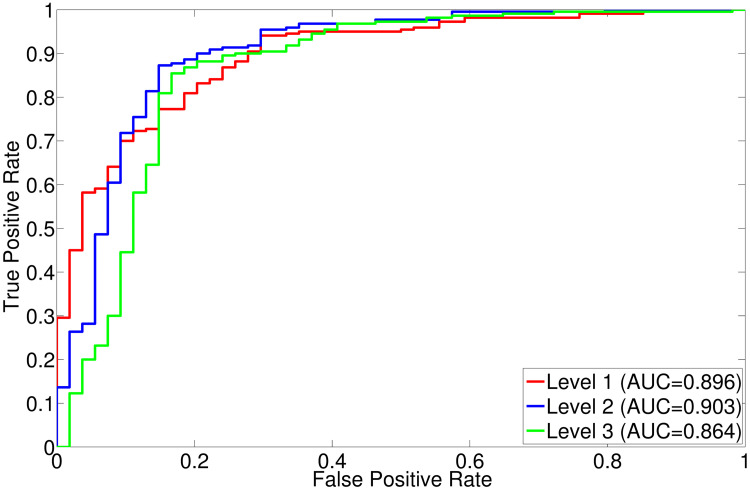
ROC curve obtained using leave-one-out cross-validation for different decomposition levels of wavelet analysis.

**Fig 9:**
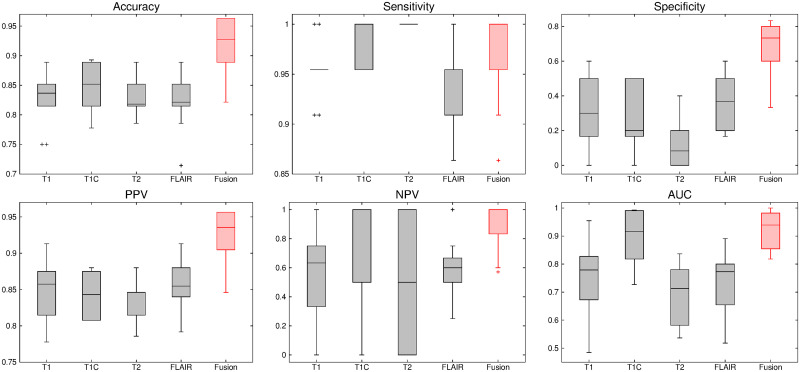
Comparative performance analysis of the proposed fusion method over individual sequences for ten-fold cross-validation.

**Fig 10:**
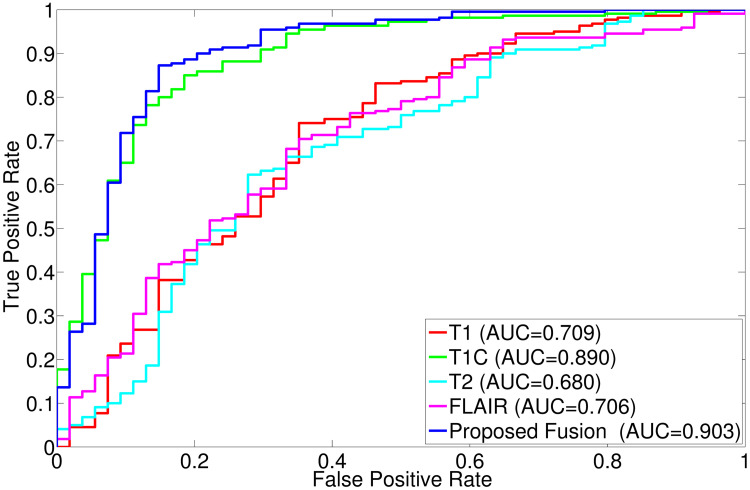
ROC curve obtained using leave-one-out cross-validation for the proposed fusion method and individual sequences.

**Fig 11:**
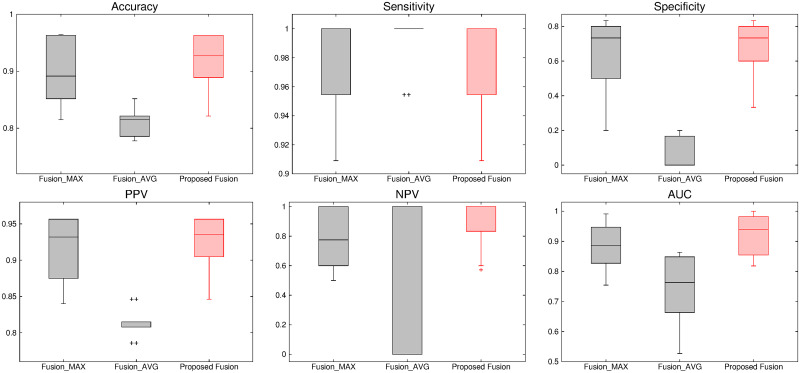
Comparative performance analysis of different fusion approaches using ten-fold cross-validation.

**Fig 12:**
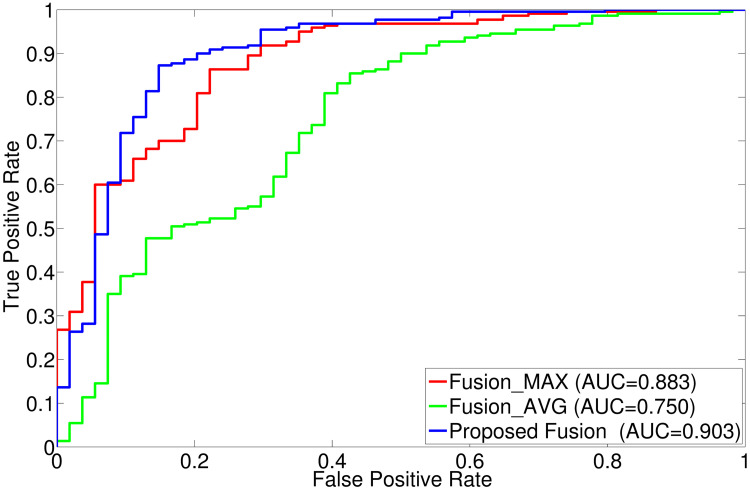
ROC curve obtained using leave-one-out cross-validation for different fusion approaches.

**Fig 13:**
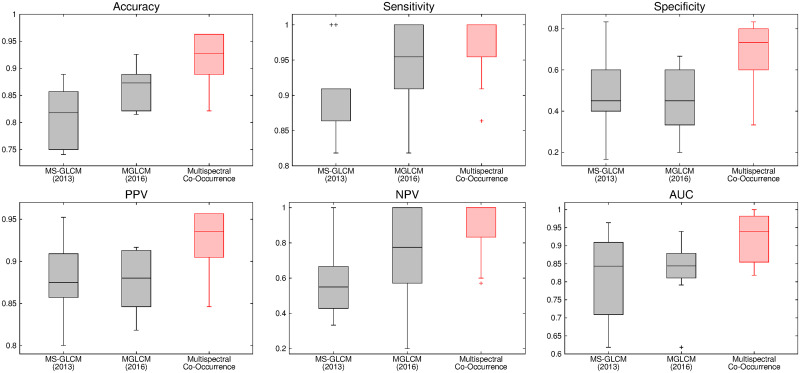
Comparative performance analysis of the proposed multispectral co-occurrence matrix and multi-scale GLCM based methods using ten-fold cross-validation.

**Fig 14:**
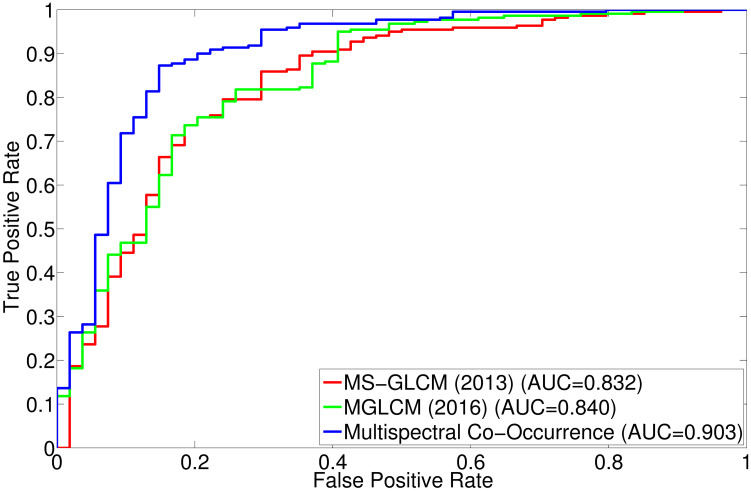
ROC curve obtained using leave-one-out cross-validation for the proposed multispectral co-occurrence matrix and multi-scale GLCM based methods.

The comparative performance analysis of different algorithms is studied using Tables 3–6, box-and-whisker plots of Figs 7, 9, 11 and 13, and ROC curves of Figs 8, 10, 12 and 14. The ROC curves in Figs 8, 10, 12 and 14 are obtained from leave-one-out cross-validation, while Figs 7, 9, 11 and 13 show the analysis for ten-fold cross-validation. Similarly, Table 3 presents the classification performance of the proposed method for leave-one-out cross-validation, while Tables 4–6 report the means, standard deviations, and p-values computed through Wilcoxon signed-rank test (one-tailed) and paired-*t* test (one-tailed) with respect to six classification indices namely, accuracy, sensitivity, specificity, PPV, NPV and AUC, for ten-fold cross-validation. Table 3 highlights the best classification indices in bold font. Similarly, in Tables 4–6, best mean indices and significant p-values, considering 95% confidence level, are marked bold, while lower, but not significant, p-values are made italics. In box-and-whisker plots of Figs 7, 9, 11 and 13, the top and bottom boundaries of each box represent upper and lower quartiles, respectively, central line represents the median, whiskers are extended to three standard deviations from the mean, and the outliers are represented by ‘+’. In the box-and-whisker plots, red color corresponds to the proposed algorithm.

**Table 3:** Performance analysis of different algorithms using leave-one-out cross-validation.

Approaches	Methods	Accuracy	Sensitivity	Specificity	PPV	NPV	AUC
Wavelet Level	Level 1	0.876	0.901	0.738	0.950	0.574	0.896
	Level 3	0.876	0.912	0.708	0.936	**0.630**	0.864
MS-GLCM (2013)		0.858	0.892	0.674	0.936	0.537	0.832
MGLCM (2016)		0.880	0.891	0.800	0.968	0.519	0.840
Fusion	Max Operator	0.891	0.913	0.773	0.955	**0.630**	0.883
	Avg Operator	0.807	0.810	0.600	0.991	0.056	0.750
Individual Sequences	T1	0.825	0.852	0.600	0.945	0.333	0.709
	T1C	0.854	0.854	0.850	0.986	0.315	0.890
	T2	0.828	0.826	**0.889**	**0.996**	0.148	0.680
	FLAIR	0.818	0.851	0.563	0.936	0.333	0.706
Proposed Algorithm		**0.901**	**0.914**	0.830	0.968	**0.630**	**0.903**

Bold font indicates the highest value.

**Table 4:** Accuracy and AUC of different algorithms for ten-fold cross-validation.

Different Approaches	Different Methods	Accuracy				AUC			
		Mean	Std Dev	Wilcoxon:p	Paired-*t*:p	Mean	Std Dev	Wilcoxon:p	Paired-*t*:p
Wavelet Level	Level 1	0.895	5.48E-2	*5.74E-2*	*1.205E-1*	0.913	6.53E-2	*1.93E-1*	*3.70E-1*
	Level 3	0.877	6.09E-2	**2.16E-2**	**2.04E-2**	0.875	1.04E-1	**1.79E-2**	**3.16E-2**
MS-GLCM (2013)		0.818	5.58E-2	**2.50E-3**	**1.35E-5**	0.822	1.12E-1	**2.50E-3**	**1.87E-3**
MGLCM (2016)		0.850	6.36E-2	**1.78E-2**	**1.57E-2**	0.833	8.94E-2	**1.42E-2**	**8.30E-3**
Fusion	Max Operator	0.894	6.12E-2	*1.73E-1*	*1.86E-1*	0.887	8.24E-2	**7.58E-3**	**1.05E-2**
	Avg Operator	0.810	2.69E-2	**2.52E-3**	**1.07E-4**	0.742	1.10E-1	**3.44E-3**	**4.45E-4**
Individual Sequences	T1	0.825	4.56E-2	**3.79E-3**	**6.30E-4**	0.743	1.43E-1	**6.23E-3**	**2.57E-3**
	T1C	0.843	4.37E-2	**6.31E-3**	**1.22E-3**	0.897	9.25E-2	*2.03E-1*	*1.22E-1*
	T2	0.825	3.15E-2	**3.82E-3**	**2.51E-4**	0.693	1.10E-1	**2.53E-3**	**2.44E-4**
	FLAIR	0.825	5.12E-2	**3.82E-3**	**3.06E-4**	0.735	1.17E-1	**3.46E-3**	**1.11E-4**
Proposed Algorithm		**0.913**	4.86E-2	-	-	**0.920**	6.56E-2	-	-

Bold font indicates best mean values and statistically significant p-values, while italics font indicates lower, but not significant, p-values.

**Table 5:** Sensitivity and specificity of different algorithms for ten-fold cross-validation.

Different Approaches	Different Methods	Sensitivity				Specificity			
		Mean	Std Dev	Wilcoxon:p	Paired-*t*:p	Mean	Std Dev	Wilcoxon:p	Paired-*t*:p
Wavelet Level	Level 1	0.960	5.00E-2	*2.25E-1*	*1.72E-1*	0.633	1.04E-1	*3.42E-1*	*3.15E-1*
	Level 3	0.936	5.34E-2	**2.06E-2**	**1.24E-2**	0.630	1.45E-1	*1.43E-1*	*2.07E-1*
MS-GLCM (2013)		0.900	6.36E-2	**9.78E-3**	**7.48E-3**	0.487	2.29E-1	*7.50E-2*	*5.86E-2*
MGLCM (2016)		0.945	6.36E-2	*2.38E-1*	*2.14E-1*	0.457	1.78E-1	**1.25E-2**	**1.03E-2**
Fusion	Max Operator	0.960	3.35E-2	**1.36E-2**	*1.78E-1*	0.623	2.49E-1	*3.43E-1*	*3.27E-1*
	Avg Operator	0.991	1.92E-2	8.82E-1	8.78E-1	0.073	9.53E-2	**2.47E-3**	**1.94E-5**
Individual Sequences	T1	0.955	3.03E-2	*1.28E-1*	*1.39E-1*	0.303	2.27E-1	**1.40E-2**	**6.62E-3**
	T1C	0.982	2.35E-2	7.75E-1	8.28E-1	0.267	1.96E-1	**3.96E-3**	**3.59E-4**
	T2	**1.00**	0.00	9.67E-1	9.67E-1	0.113	1.36E-1	**2.52E-3**	**2.89E-5**
	FLAIR	0.941	3.74E-2	*5.10E-2*	*8.40E-2*	0.357	1.71E-1	**3.82E-3**	**3.81E-4**
Proposed Algorithm		0.968	4.82E-2	-	-	**0.667**	1.83E-1	-	-

Bold font indicates best mean values and statistically significant p-values, while italics font indicates lower, but not significant, p-values.

**Table 6:** PPV and NPV of different algorithms for ten-fold cross-validation.

Different Approaches	Different Methods	PPV				NPV			
		Mean	Std Dev	Wilcoxon:p	Paired-*t*:p	Mean	Std Dev	Wilcoxon:p	Paired-*t*:p
Wavelet Level	Level 1	0.913	2.84E-2	*2.23E-1*	*2.59E-1*	0.827	1.96E-1	*6.90E-2*	*6.85E-2*
	Level 3	0.912	3.53E-2	*1.13E-1*	*1.38E-1*	0.740	1.93E-1	**2.11E-2**	**1.20E-2**
MS-GLCM (2013)		0.880	5.10E-2	**2.34E-2**	**3.66E-2**	0.599	2.40E-1	**5.40E-3**	**1.18E-3**
MGLCM (2016)		0.878	3.46E-2	**1.09E-2**	**6.90E-3**	0.742	2.73E-1	*9.15E-2*	*1.27E-1*
Fusion	Max Operator	0.915	4.86E-2	*3.00E-1*	*3.40E-1*	0.765	1.97E-1	*6.81E-2*	*5.70E-2*
	Avg Operator	0.814	2.04E-2	**2.52E-3**	**2.44E-5**	0.350	4.74E-1	**8.38E-3**	**2.23E-3**
Individual Sequences	T1	0.849	4.67E-2	**1.04E-2**	**5.36E-3**	0.527	3.27E-1	**5.36E-3**	**5.17E-3**
	T1C	0.810	1.12E-1	**4.65E-3**	**1.06E-2**	0.725	4.16E-1	*1.72E-1*	*1.21E-1*
	T2	0.822	2.87E-2	**2.52E-3**	**4.75E-5**	0.500	5.27E-1	**3.06E-2**	**2.76E-2**
	FLAIR	0.857	4.00E-2	**2.53E-3**	**3.04E-4**	0.603	1.95E-1	**5.40E-3**	**2.35E-3**
Proposed Algorithm		**0.924**	3.90E-2	-	-	**0.884**	1.71E-1	-	-

Bold font indicates best mean values and statistically significant p-values, while italics font indicates lower, but not significant, p-values.

The results reported in Figs 9 and 10 and Tables 3–6 establish the importance of the proposed fusion technique. From all the results reported in Fig 10 and Table 3, it is seen that the proposed algorithm, fusing all MR sequences, outperforms the methods using individual MR sequences with respect to all indices, except for specificity and PPV. For these indices, T2 yields better results than the proposed fusion method. Analyzing the sensitivity and NPV in Table 3, it is seen that T2 obtains lowest values for these indices among all the methods including individual MR sequences and the proposed algorithm. The proposed method obtains best results in 4 cases, out of total 6 classification indices, with respect to all the individual MR sequences, in case of leave-one-out cross-validation.

### 3.1 Data sets used

Two multi-sequence brain MR volume data sets namely, BRATS 2012 and BRATS 2014, are used in the current research work. Each of these data sets contains four different MR sequences namely, T1, T1C, T2 and FLAIR. All volumes are skull stripped and linearly co-registered to the corresponding T1C MR sequence. Both BRATS 2012 and BRATS 2014 data sets provide the manual segmentations and diagnostic labels (high or low grade) for tumors, done by experts. The BRATS 2012 brain tumor image data [25] is obtained from the MICCAI 2012 Challenge on Multimodal Brain Tumor Segmentation (www.imm.dtu.dk/projects/BRATS2012) organized by B. Menze, A. Jakab, S. Bauer, M. Reyes, M. Prastawa, and K. Van Leemput. The BRATS 2014 tumor image data [25] is obtained from the MICCAI 2014 Challenge on Multimodal Brain Tumor Segmentation (www.braintumorsegmentation.org/) organized by K. Farahani, M. Reyes, B. Menze, E. Gerstner, J. Kirby and J. Kalpathy-Cramer. The challenge database contains fully anonymized images from the following Institutions: ETH Zurich, University of Bern, University of Debrecen, and University of Utah and publicly available images from the Cancer Imaging Archive. BRATS 2012 data set contains 30 real brain MR volumes, in which 20 are high grade and remaining 10 are low grade. BRATS 2014 database has significantly enlarged training data set obtained from the NIH Cancer Imaging Archive. It contains 200 real high grade and 44 real low grade brain MR volumes. In order to analyze the performance of the proposed algorithm, these two data sets are merged, resulting in total 220 high grade and 54 low grade brain MR volumes for each MR sequence.

### 3.2 Optimum value of wavelet decomposition level

The 3-D wavelet transform generates one approximation and seven detail subbands at each level. In case of dyadic wavelet transform, the approximation part is iteratively decomposed as the decomposition level is increased. Hence, if an input volume is decomposed upto *L*th level, total 7*L* number of detail subbands are generated. In order to find out the optimum value of decomposition level *L*, experiments are carried out on several brain MR volumes by varying *L* = 1 to 3. To compare the performance of the proposed method at different wavelet decomposition levels, Figs 7 and 8 report box-and-whisker plots and ROC curve, respectively, while Tables 3–6 depict the classification results with respect to different indices. The methods with decomposition levels of one to three are named as “Level 1”, “Level 2”, and “Level 3”, respectively, in Figs 7 and 8. As shown in Fig 7, the performance of the proposed algorithm is better when the wavelet decomposition level *L* = 2 than that of other levels using ten-fold cross-validation, irrespective of the classification indices used. The improvement of classification performance can also be seen in ROC curve of Fig 8 obtained from leave-one-out cross-validation strategy. Since the proposed tumor classification algorithm uses wavelet decomposition level upto two, the proposed algorithm in Tables 3–6 corresponds to the method “Level 2” of Figs 7 and 8.

In leave-one-out cross-validation, it is seen in Table 3 that the proposed algorithm performs better than the methods of “Level 1” and “Level 3”, irrespective of the classification indices used. In case of ten-fold cross-validation, as shown in Tables 4–6, the proposed method also achieves best mean values for all the classification indices, at decomposition level 2. There are total 24 comparisons with the methods “Level 1” and “Level 3”, in terms of p-values, computed through Wilcoxon signed-rank test and paired-*t* test, for all six indices. Out of total 24 cases, the proposed method at *L* = 2 performs significantly better in 8 cases and attains lower, but not significant, p-values in remaining 16 cases, as shown in Tables 4–6. So, it can be concluded that the classification performance of the proposed method at wavelet decomposition level *L* = 2 is better than that of other levels, irrespective of the quantitative indices and experimental setup used. Hence, each volume is decomposed upto level *L* = 2 in the current study.

### 3.3 Importance of fusion over individual sequences

This section establishes the importance of information fusion from multiple MR sequences over individual ones. The classification performance is analyzed using ten-fold cross-validation as well as leave-one-out cross-validation. In case of ten-fold cross-validation, it is seen in Fig 9 and Tables 4–6 that the method using single T1C sequence performs better than the methods using other MR sequences namely, T1, T2 and FLAIR, with respect to all the indices, except in three cases. The T2 sequence is better with respect to sensitivity, while FLAIR sequence obtains highest mean values for specificity and PPV. For leave-one-out cross-validation (Table 3), it is seen that different MR sequences produce highest values for different classification indices. In case of accuracy, sensitivity, and AUC, the T1C sequence yields better results, while T2 sequence gives highest specificity and PPV values and T1 and FLAIR are having best result in NPV. Therefore, it can be concluded that individual MR sequence is not sufficient to characterize all the subregions of tumors, in turn, poorly determines the malignancy state of entire tumor. A combination of these sequences may provide a considerable amount of information for revealing the underlying tumor grade.

Analyzing the box plots of Fig 9, it is observed that the proposed method generates highest median values with respect to accuracy, specificity, PPV, and AUC. For remaining two indices namely, sensitivity and NPV, the proposed method achieves highest median values, which are also obtained by some of the individual MR sequences such as T1C and T2. As depicted in Tables 4–6, the performance of the proposed method, in case of ten-fold cross-validation, is improved with respect to all individual sequences irrespective of the quantitative indices used. However, the method using individual T2 sequence produces highest sensitivity value of 1.0 and lower specificity values. It reveals that the false positive count is very high, while the false negative count is zero, indicating the overall poor performance of the method using T2 sequence. Therefore, the proposed method performs significantly better than any individual MR sequences namely, T1, T1C, T2 and FLAIR, in 36 cases, out of total 48 comparisons, while it obtains lower, but not significant, p-values in 8 cases. The AUC values of the proposed algorithm and the methods using individual MR sequences, as shown in Fig 10, indicate that the proposed method provides significantly better performance than the methods using T1, T1C, T2, and FLAIR modalities. All these results indicate the importance of the proposed fusion method over the use of single MR sequence with respect to six classification indices.

### 3.4 Effectiveness of texture gradient based wavelet fusion

The proposed method employs texture gradient based fusion method to integrate multi-sequence MR volumes namely, T1, T1C, T2 and FLAIR. It uses the texture information content of different sequences to fuse the wavelet coefficients for each voxel within the region of interest. Figs 11 and 12 and Tables 3–6 present the effectiveness of the proposed fusion algorithm embedded in the method, over two other fusion approaches done by maximum and average operators. In this section, these two methods are named as Fusion_MAX and Fusion_AVG.

In leave-one-out cross-validation strategy, the proposed tumor classification algorithm obtains highest values for all the indices, with respect to both Fusion_MAX and Fusion_AVG methods, as shown in Table 3. However, Fusion_AVG method performs better than the proposed method with respect to PPV. For ten-fold cross-validation, the proposed method attains higher mean values than that of methods using fusion with these two operators for all classification metrics used, except sensitivity. Therefore, the proposed method performs significantly better than the fusion with average operator in 10 cases, out of 12 cases, while it yields lower p-values compared to the method using fusion with maximum operator in all cases, irrespective of quantitative indices used. In addition, the median values of the proposed algorithm are higher than that of the methods Fusion_MAX and Fusion_AVG for all the classification indices, as shown in Fig 11, except specificity with respect to Fusion_MAX method. In this case, the proposed algorithm and the Fusion_MAX method obtain same median specificity value of 0.733. However, the mean specificity value of the proposed algorithm is better than that of the Fusion_MAX method as shown in Table 5. Analyzing the AUC values computed through leave-one-out cross-validation, presented in Fig 12, it is seen that the proposed fusion method outperforms both Fusion_MAX and Fusion_AVG methods. The better performance of the proposed tumor gradation method is achieved due to the fact that the texture content, which is effective for characterizing tumor type, is used to obtain most desirable information at each spatial position of each wavelet subband for a specific MR sequence.

### 3.5 Importance of multispectral co-occurrence

The proposed algorithm computes the co-occurrence of detail wavelet coefficients from multiple subbands and extracts Haralick features from those multispectral co-occurrence matrices. In this section, the performance of the proposed algorithm is compared with that of two existing multi-scale GLCM based methods namely, MS-GLCM (2013) [12] and MGLCM (2016) [17]. In these methods, the features are extracted for each of T1, T1C, T2, and FLAIR modalities and then concatenated to obtain the final feature vector. In order to keep the same experimental background, seven Haralick features are considered and the SVM with linear kernel is used as classifier for MS-GLCM (2013) [12] and MGLCM (2016) [17], as done for the proposed tumor gradation algorithm. Figs 13 and 14 and Tables 3–6 present the improvement of the classification performance using multispectral co-occurrence matrices over two related methods namely, MS-GLCM (2013) and MGLCM (2016). In Figs 13 and 14, the proposed algorithm using multispectral co-occurrence matrices is named as “Multispectral Co-Occurrence”.

From the results reported in Tables 4–6, it is observed that the proposed algorithm achieves better classification indices with respect to both the methods for ten-fold cross-validation strategy. In addition, analyzing the classification results of Table 3 for leave-one-out cross-validation, it is seen that the proposed tumor gradation algorithm performs better than both MS-GLCM (2013) and MGLCM (2016) with respect to all indices, except PPV, in which both the proposed method and MGLCM (2016) achieve same value of 0.968182. Out of total 24 cases of ten-fold cross-validation, the proposed method obtains significant p-values in 18 cases and lower, but not significant, p-values in remaining 6 cases, irrespective of the classification indices used, as shown in Tables 4–6. Analyzing the box plots of Fig 13 for ten-fold cross-validation, it is observed that the proposed method yields higher median values compared to both MS-GLCM (2013) and MGLCM (2016), for all the classification indices. The better performance of the proposed algorithm can also be seen with respect to ROC curve of Fig 14, obtained using leave-one-out cross-validation strategy. The AUC value of the proposed method is 0.903, which is significantly higher than that of the methods MS-GLCM (2013) and MGLCM (2016), as shown in Fig 14. The significantly better performance of the proposed algorithm with respect to both MS-GLCM (2013) and MGLCM (2016) is achieved due to the fact that the proposed algorithm takes benefit from multiresolution wavelet analysis. Moreover, the proposed method considers multispectral co-occurrence matrices, rather than restricting itself within single wavelet subband, to capture the image content of all high frequency component of the image. It helps to derive the co-occurrence of the pair of wavelet coefficients from multiple subbands.

## 4 Conclusion

This paper presents a new algorithm for the gradation of brain tumor, using conventional MR sequences. The contribution of this work is mainly three-fold namely,

1. development of a wavelet based fusion method to integrate complementary information of multiple MR sequences;
2. defining a new spatial relationship among the pair of wavelet coefficients of different wavelet subbands, called multispectral co-occurrence of wavelet coefficients; and
3. demonstrating the performance of the proposed tumor gradation algorithm, along with a comparison with other related methods.

The proposed fusion method consolidates the wavelet coefficients of each of the four MR sequences namely, T1, T1C, T2 and FLAIR, based on the texture information content. It is observed that the fusion of different MR sequences boosts the performance of the proposed method significantly over that of individual MR sequences. The feature descriptor, obtained from the proposed multispectral co-occurrence of wavelet coefficients, effectively extracts the important detail information, obtained across the wavelet subbands. Finally, the effectiveness of the proposed algorithm is evaluated by both leave-one-out and ten-fold cross-validation strategies, on a set of 274 real brain MR volumes, obtained from BRATS 2012 and BRATS 2014 data sets.

## 5 Appendix

### 5.1 Computation of multispectral co-occurrence matrices

**Fig 15:**
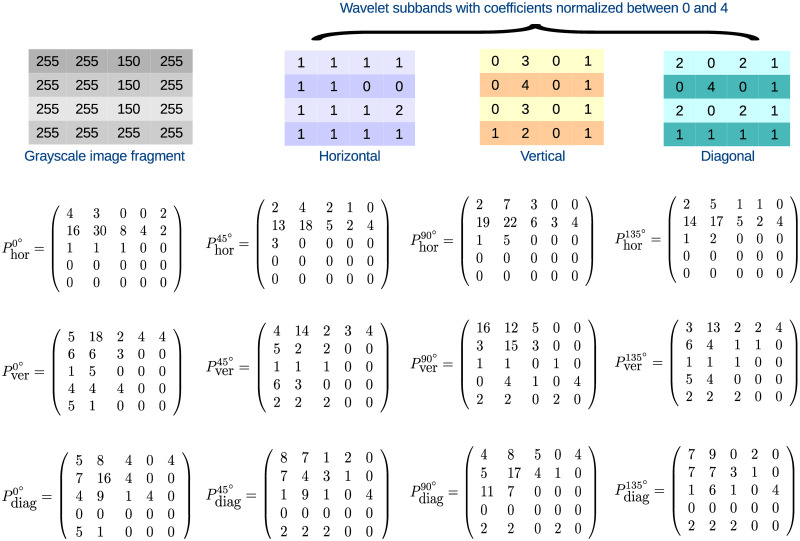
Calculation of multispectral co-occurrence matrix for all detail wavelet subbands at four directions.

In this section, the computation of proposed multispectral co-occurrence matrix is elaborated with the help of an example image. Fig 15 shows a 4 × 4 fragment region of the top gray-scale image of Fig 2 and the wavelet decompositions of the corresponding fragment region. In this example, the wavelet coefficients of the detail subbands namely, horizontal, vertical and diagonal, are normalized in the range between 0 and 4. The multispectral co-occurrence matrix is then computed for each wavelet detail subband at each direction namely, horizontal (0°), right-diagonal (45°), vertical (90°) and left-diagonal (135°). The first four matrices namely, $P_{\text{hor}}^{0^{{}^{\circ}}}$, $P_{\text{hor}}^{45^{{}^{\circ}}}$, $P_{\text{hor}}^{90^{{}^{\circ}}}$ and $P_{\text{hor}}^{135^{{}^{\circ}}}$, portray four multispectral co-occurrence matrices with respect to horizontal subband, which represent the spatial dependency of inter- and intra-subband wavelet coefficients at the directions of horizontal, right-diagonal, vertical, and left-diagonal, respectively. Similarly, the multispectral co-occurrence matrices with respect to vertical and diagonal subbands, denoted as $P_{\text{ver}}^{\theta^{{}^{\circ}}}$ and $P_{\text{diag}}^{\theta^{{}^{\circ}}}$, respectively, are also depicted in Fig 15. In this example, two neighboring points are separated by a distance of 1. In order to determine the element in the (*i*, *j*) position of the matrix $P_{\text{ver}}^{0^{{}^{\circ}}}$, for example, the values of three terms are added, as the number of wavelet subbands is 3. These three terms are $c_{V_{H}}$, $c_{V_{V}}$, and $c_{V_{D}}$, described as follows:

-
$c_{V_{H}}$ denotes the number of times the wavelet coefficient of value *i* in vertical wavelet subband and that of value *j* in horizontal subband, occurred horizontally adjacent to each other with respect to spatial coordinates within the respective subband;
-
$c_{V_{V}}$ denotes the total number of times two wavelet coefficients of values *i* and *j* occurred horizontally adjacent to each other in the vertical wavelet subband; and
-
$c_{V_{D}}$ denotes the number of times the wavelet coefficient of value *i* in vertical wavelet subband and that of value *j* in diagonal subband, occurred horizontally adjacent to each other with respect to spatial coordinates within the respective subband.

For example, the element in the (0, 0) position of the matrix $P_{\text{ver}}^{0^{{}^{\circ}}}$ is 5, as shown in Fig 15. To determine this number, the values of $c_{V_{H}}$, $c_{V_{V}}$, and $c_{V_{D}}$, corresponding to the (0, 0) position of $P_{\text{ver}}^{0^{{}^{\circ}}}$, are to be computed. Since there is no pair of wavelet coefficients of value 0 occurred horizontally in vertical subband, the value of $c_{V_{V}}=0$. In order to compute $c_{V_{D}}$, let us consider the coordinate position of (2, 2) in vertical subband. If the neighbors of (2, 2) position of diagonal subband are considered with the wavelet coefficient at position (2, 2) of vertical subband as the center of the neighborhood, then the corresponding neighborhood region becomes

$$\begin{array}{c} \left(\begin{array}{cccc} \ldots & \ldots & \ldots & \ldots \\ \ldots & 4 & 0 & 1\\ \ldots & 0 & 0 & 1\\ \ldots & 1 & 1 & 1 \end{array}\right) \end{array}$$

Here, two wavelet coefficients of value 0 occur horizontally adjacent to each other. Similarly, considering the coordinate positions (0, 0), (0, 2), and (2, 0) in vertical subband with respect to diagonal subband, a pair of wavelet coefficients of value 0, adjoined to each other horizontally, is found for each position. Therefore, the value of $c_{V_{D}}=4$. Again, there exists only one pair of wavelet coefficients of value 0, when computing multispectral co-occurrence of wavelet coefficients for vertical subband with respect to horizontal subband. Hence, $c_{V_{H}}=1$. So, the entry of the position (0, 0) of the matrix $P_{\text{ver}}^{0^{{}^{\circ}}}$ is $c_{V_{H}}+c_{V_{V}}+c_{V_{D}}=5$. In a similar way, the other elements of this matrix $P_{\text{ver}}^{0^{{}^{\circ}}}$, and the elements of other multispectral co-occurrence matrices for different wavelet subbands and different angular relationships of adjacent neighbors can be computed, as shown in Fig 15.

### 5.2 Co-occurrence matrices for volumetric data

**Fig 16:**
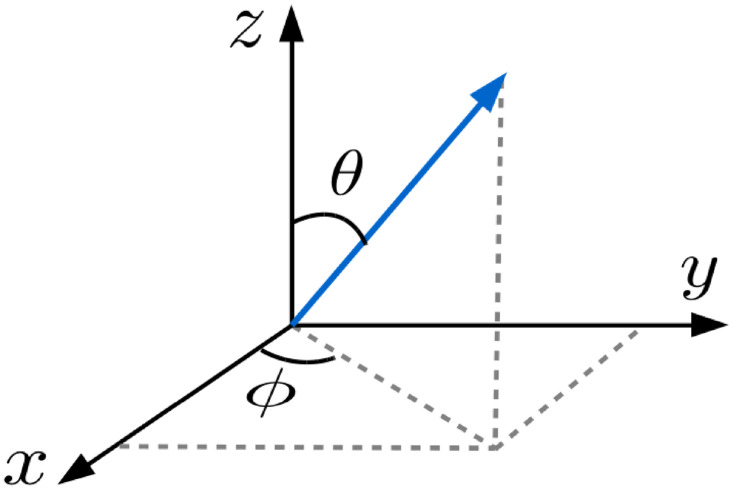
Illustration of the displacement vector in 3-D space.

Let, *θ* be the angle between the vector (blue line) and the *z*-axis, and *φ* be the angle between the projection of the vector on *x* − *y* plane and the *x*-axis, as depicted in Fig 16. Since the co-occurrence matrix is computed over volumetric data, there are 26 different possible combinations of the displacement vector. Among them, only 13 displacement vectors, $\Delta \tilde{r}=\left(dx,dy,dz\right)$ are different, which are shown in Table 7 along with the corresponding values of *θ* and *φ*. However, for the simplification of computation, it is usually set as one voxel in distance and it, thus, yields only 13 different types of configurations of wavelet coefficient pairs at 3-D space.

**Table 7:** Displacement vector for multispectral co-occurrence matrices.

Direction (*θ*, *φ*)	Displacement Vector
(0°, −)	(0, 0, 1)
(45°, 0°)	(1, 0, 1)
(45°, 45°)	(1, 1, 1)
(45°, 90°)	(0, 1, 1)
(45°, 135°)	(-1, 1, 1)
(90°, 0°)	(1, 0, 0)
(90°, 45°)	(1, 1, 0)
(90°, 90°)	(0, 1, 0)
(90°, 135°)	(-1, 1, 0)
(135°, 0°)	(1, 0, -1)
(135°, 45°)	(1, 1, -1)
(135°, 90°)	(0, 1, -1)
(135°, 135°)	(-1, 1, -1)

### 5.3 Classification indices

The basic idea of performing a diagnostic test is to ensure about a particular disease such that treatment planning can be done properly. The validity of the diagnostic test can be assessed quantitatively by different classification indices namely, accuracy, sensitivity, specificity, positive predictive value and negative predictive value, with the help of ground truth information.

#### 5.3.1 Accuracy

The classification accuracy is defined as the number of correct predictions made divided by the total number of predictions made, given by

$$\begin{array}{c} \text{accuracy}=\frac{\text{TP}+\text{TN}}{\text{TP}+\text{TN}+\text{FP}+\text{FN}}; \end{array}$$ (4)

where TP denotes true positive, TN is true negative, FP denotes false positive, and FN presents false negative.

#### 5.3.2 Sensitivity

Sensitivity is the ability of a test to correctly classify an individual as diseased. It is defined as the probability of being a test positive when the disease is present, which is given by

$$\begin{array}{c} \text{sensitivity}=\frac{\text{TP}}{\text{TP}+\text{FN}}. \end{array}$$ (5)

#### 5.3.3 Specificity

Specificity is the ability of a test to correctly classify an individual as disease-free. It is defined as the probability of being a test negative when the disease is absent, which is given as follows:

$$\begin{array}{c} \text{specificity}=\frac{\text{TN}}{\text{TN}+\text{FP}}. \end{array}$$ (6)

#### 5.3.4 Positive predictive value

The positive predictive value (PPV) is the percentage of patients with a positive test who actually have the disease. It indicates how many of test positives are true positives; and if this number is higher, then it suggests that a new test is doing as good as gold standard. The PPV is defined as the probability that a patient having disease when test is positive, defined as follows:

$$\begin{array}{c} \text{PPV}=\frac{\text{TP}}{\text{TP}+\text{FP}}. \end{array}$$ (7)

#### 5.3.5 Negative predictive value

The negative predictive value (NPV) is the percentage of patients with a negative test who do not have the disease. It indicates how many of negative tests are true negative. The NPV is defined as the probability of a patient not having disease when the test is negative, which is given by

$$\begin{array}{c} \text{NPV}=\frac{\text{TN}}{\text{TN}+\text{FN}}. \end{array}$$ (8)

The higher values of different classification indices namely, accuracy, sensitivity, specificity, PPV and NPV, indicate the better classification performance.

#### 5.3.6 Area under curve

The area under receiver operating characteristic (ROC) curve or simply, area under curve (AUC), is a popular performance measure for classification problem at various test cutoff points. It evaluates and compares the classification rules when one cannot decide a priori what classification threshold will be used. The diagnostic performance of a test or the accuracy of a test to discriminate the diseased cases from normal cases is evaluated using ROC curve analysis. The ROC curve displays all possible cut-off points, and one can read the optimal cut-off for correctly identifying diseased or non-diseased subjects. The ROC curve is graphical display of sensitivity, also known as true positive rate (TPR) on *y*-axis and (1—specificity) or false positive rate (FPR) on *x*-axis for varying cut-off points of test values.

The AUC is an effective way to summarize the overall diagnostic accuracy of the test. The values of AUC lie within 0.5 and 1. A value of 0.5 for AUC indicates that the ROC curve will fall on the line of equality and hence, it suggests that the diagnostic test has no discriminatory ability. The ROC curve above this diagonal line, that is, the value of AUC close to 1, represents better performance of the test. Furthermore, the AUC value of 1 indicates that the diagnostic test is perfect in differentiating the diseased from non-diseased subjects. This implies both sensitivity and specificity are one and both errors namely, false positive and false negative, are zero.
